# First Manic Episode After a Loss Experience: A Case of Funeral Mania

**DOI:** 10.1192/j.eurpsy.2025.1041

**Published:** 2025-08-26

**Authors:** U. Altunoez, B. Wolff, I. B. Guevercin, A.-C. Hergott

**Affiliations:** 1 Department of General Psychiatry and Psychotherapy, KRH Wunstorf Psychiatric Hospital, Wunstorf, Germany

## Abstract

**Introduction:**

There is a paucity of research on the relationship between bereavement and the onset of bipolar disorder, especially in connection with manic episodes. While some case reports delivered preliminary data, they are insufficient to determine whether the stress response to a loss triggers the first signs of the disorder, or if manic symptoms arise in individuals with pre-existing mood instabilities. Overall, the predictors and prodromal characteristics for the development of a manic episode following a loss remain unclear. Moreover, there are no follow-up case studies to evaluate the long-term outcomes of these patients after the first manic episode.

**Objectives:**

In this presentation, we will discuss the case of a woman who experienced her first manic episode immediately after the death of her son and present the one-year follow-up process to provide some experience in the psychopharmacological and psychotherapeutic treatment of these patients.

**Methods:**

Here we will present a case from a psychiatric-psychotherapeutic hospital in Lower Saxony, Germany, in a comprehensive way.

**Results:**

A 43-year-old woman was referred to the psychiatric department three weeks after the sudden death of her 15-year-old son due to an undiscovered congenital heart defect. She presented with chest pain, anxiety, sleep disorders, and referential psychotic thoughts. In the intensive psychiatric ward, she exhibited mood swings, sexual disinhibition, agitation, and aggressive behaviors towards staff. She engaged in excessive spending and refused to take her medications, Risperidone and Quetiapine. Her history included one depressive episode, successfully treated with psychotherapy and medication, but no previous manic episodes. Additionally, one brother had committed suicide, and another brother and her father had died from congenital heart conditions. We applied an intensive dynamic, systemic approach involving her family members to create a supportive environment for processing grief while managing her manic symptoms. After insisting on discharge after one month, she stopped her medication, leading to another intensive ward treatment due to aggression. During this phase, we resumed the same medications and intensive psychotherapy, resulting in stabilization. She later entered a psychosomatic treatment program, where she discontinued her antipsychotic medication and focused on her grief. Six months after this treatment, just before the anniversary of her son’s death, she fell into a deep depression with suicidal thoughts and was referred to an open ward, where she was successfully treated with lithium and supportive psychotherapy. All somatic examinations and brain MRI scans were normal.

**Conclusions:**

Bereavement can manifest in various ways, and in a small number of patients, it can trigger the first manic episode of bipolar disorder. Further detailed follow-up research is needed to optimize the diagnostic and treatment processes for these patients.

**Disclosure of Interest:**

None Declared

